# Supplementary material for: Empirical methods for controlling false positives and estimating confidence in ChIP-Seq peaks
Source: BMC Bioinformatics. 2008 Dec 5;9:523. doi: 10.1186/1471-2105-9-523 (PMC2628906; doi:10.1186/1471-2105-9-523)
Supplement: Additional File 1 — A variety of html documents from the USeq web site detailing the available applications, their best usage, output file type descriptions, command line menus, etc. [file 1471-2105-9-523-S1.zip › USeqUserGuides/JavaDoc.zip!/JavaDoc/bioroot/Organization.html]

Organization


|  |  |  |
| --- | --- | --- |
| **Overview** \| **Package** \| **Class** \| **Use** \| **Tree** \| **Deprecated** \| **Index** \| **Help** | |  |
| **PREV CLASS**   **NEXT CLASS** | **FRAMES**    **NO FRAMES**     **All Classes** |  |
| SUMMARY: NESTED \| FIELD \| CONSTR \| METHOD | DETAIL: FIELD \| CONSTR \| METHOD |  |


---


## bioroot Class Organization

```
java.lang.Object
  bioroot.Organization
```

---

``` public class Organization extends java.lang.Object ```

Container for holding info about Organization membership.
Used by reagents and the userBean

---

| **Field Summary** | |
| --- | --- |
| `static org.apache.log4j.Logger` | `log` |


**Constructor Summary**

`Organization(DBUtil bioRoot,
int labGroupId)`


| **Method Summary** | |
| --- | --- |
| `int` | `getId()` |
| `java.lang.String` | `getName()` |
| `void` | `loadOrganizationInfo(DBUtil bioRoot, int labGroupId)` |
| `void` | `setId(int id)` |
| `void` | `setName(java.lang.String name)` |

**Methods inherited from class java.lang.Object**

`equals, getClass, hashCode, notify, notifyAll, toString, wait, wait, wait`

**Field Detail**

### log

```
public static org.apache.log4j.Logger log
```


**Constructor Detail**

### Organization

```
public Organization(DBUtil bioRoot,
                    int labGroupId)
```


**Method Detail**

### loadOrganizationInfo

```
public void loadOrganizationInfo(DBUtil bioRoot,
                                 int labGroupId)
```

---


### getId

```
public int getId()
```

---


### setId

```
public void setId(int id)
```

---


### getName

```
public java.lang.String getName()
```

---


### setName

```
public void setName(java.lang.String name)
```


---


|  |  |  |
| --- | --- | --- |
| **Overview** \| **Package** \| **Class** \| **Use** \| **Tree** \| **Deprecated** \| **Index** \| **Help** | |  |
| **PREV CLASS**   **NEXT CLASS** | **FRAMES**    **NO FRAMES**     **All Classes** |  |
| SUMMARY: NESTED \| FIELD \| CONSTR \| METHOD | DETAIL: FIELD \| CONSTR \| METHOD |  |


---
